# Site-Specific DC Surface Signatures Influence CD4^+^ T Cell Co-stimulation and Lung-Homing

**DOI:** 10.3389/fimmu.2019.01650

**Published:** 2019-07-18

**Authors:** David Pejoski, Marie Ballester, Floriane Auderset, Maria Vono, Dennis Christensen, Peter Andersen, Paul-Henri Lambert, Claire-Anne Siegrist

**Affiliations:** ^1^Department of Pathology and Immunology, Faculty of Medicine, University of Geneva, Geneva, Switzerland; ^2^World Health Organization Collaborating Center for Vaccine Immunology, Faculty of Medicine, University of Geneva, Geneva, Switzerland; ^3^Center for Vaccine Research, Statens Serum Institut, Copenhagen, Denmark; ^4^Department of Immunology and Microbiology, University of Copenhagen, Copenhagen, Denmark

**Keywords:** CD11b^+^ dendritic cells, lung CD4^+^ T cells, lung homing, tissue imprinting, costimulation, vaccination route

## Abstract

Dendritic cells (DCs) that drain the gut and skin are known to favor the establishment of T cell populations that home to the original site of DC-antigen (Ag) encounter by providing *soluble* “imprinting” signals to T cells in the lymph node (LN). To study the induction of lung T cell-trafficking, we used a protein-adjuvant murine intranasal and intramuscular immunization model to compare *in vivo*-activated Ag^+^ DCs in the lung and muscle-draining LNs. Higher frequencies of Ag^+^ CD11b^+^ DCs were observed in lung-draining mediastinal LNs (MedLN) compared to muscle-draining inguinal LNs (ILN). Ag^+^ CD11b^+^ MedLN DCs were qualitatively superior at priming CD4^+^ T cells, which then expressed CD49a and CXCR3, and preferentially trafficked into the lung parenchyma. CD11b^+^ DCs from the MedLN expressed higher levels of surface podoplanin, Trem4, GL7, and the known co-stimulatory molecules CD80, CD86, and CD24. Blockade of specific MedLN DC molecules or the use of sorted DC and T cell co-cultures demonstrated that DC surface phenotype influences the ability to prime T cells that then home to the lung. Thus, the density of dLN Ag^+^ DCs, and DC *surface* molecule signatures are factors that can influence the output and differentiation of lung-homing CD4^+^ T cells.

## Introduction

Protection against major human respiratory pathogens, such as *Mycobacterium tuberculosis* (MTB)*, Bordetella pertussis* or influenza viruses would benefit from the persistence of lung effector T cells and/or the rapid reactivation of lung resident memory T cells. Yet, the global mechanisms that initiate enhanced trafficking to the airways are not well-characterized. Most vaccines administered via the parenteral route elicit inferior airway mucosa immune responses compared to those administered via the intranasal (i.n.) or intrapulmonary routes, although examples of the contrary exist, reviewed in (1, 2). Therefore, a better understanding of how lung-trafficking T cells are induced *in vivo* could guide the rational design of vaccine formulations (3) and immunotherapeutic strategies (4) that provide the desired tissue “imprinting” signals (5) to T lymphocytes.

Cell surface profiles of pulmonary T cells have been defined previously in mice and humans, and include elevated expression of one or more of the receptors: BLT-1, CCR1, CCR3, CCR4, CCR5, CCR6, CCR8, CXCR3, CXCR4, CXCR6, CD69, CD103, LFA-1, PSLG-1, or VLA-1 (3, 6–13). The majority of these markers have not been proven to mediate lung-trafficking or retention *per se*, but serve as surrogate markers given their increased expression by lung T cells. Among them, CXCR3 and CD49a (integrin α1 subunit) are being used increasingly as lung homing T cell markers. CXCR3 mediates chemotaxis toward inflamed tissues (14) including the lung (15). During influenza infection, the CXCR3-CXCL10 (IP-10) axis appears critical for the recruitment of pulmonary CD8^+^ T cells that control the infection (16). Similarly, MTB infection or immunization can induce protective CXCR3^+^ CD4^+^ T cells that readily enter the lung parenchyma (17, 18). CD49a appears to allow more selective trafficking into respiratory and reproductive tissues (3, 6), supporting an important role for adhesion molecules in the control of tissue tropism (11). CD49a has been additionally described to play a role in lung tissue retention in humans, alongside other molecules, such as CD69 (12).

Dendritic cells (DC) mediate critical roles in shaping T cell responses—including tissue-specific T cell imprinting (19, 20). For example, *soluble* vitamin A and D derivatives produced by intestine or dermis-draining DCs, respectively upregulate gut and skin homing markers on murine T cells (21–23), and similar imprinting mechanisms are likely to be at play in humans (24–28). Less is known about the contribution of tissue-specific DC *surface* markers to T cell tissue-homing. In general, DC stimulatory signals influence many features of newly primed T cells, reviewed in (29), including differentiation into effector vs. memory cells (30) or various T-helper (Th) subsets (31). For instance, cell surface TCR and CD28-mediated signaling rapidly induce tissue-adhesion molecules, such as P- and E-selectin on T cells (32). Furthermore, binding of DC-expressed CD80 with CTLA-4 contributes to the induction of LFA-1 on T cells, which can mediate mucosal tissue retention (33). T cell homing markers, including those that allow trafficking into the lung, may thus be modulated by surface DC:T cell interactions during priming. Initial evidence of DC-mediated imprinting of lung-homing markers on T cells has been demonstrated using murine DCs from the MedLN that were pulsed with Ag (34). The *in vitro* primed T cells expressed higher levels of CCR4 and showed an enhanced capacity to migrate into the lung compared to cells primed by DCs from other LNs.

In the mouse lung, reports have described two major conventional (cDC) DC subsets distinguished by their surface phenotype (35, 36). The airways are rich in CD103^+^ type 1 cDC (cDC1) which extend their dendrites into alveolar spaces, and CD11b^+^ type 2 cDC (cDC2) are usually present in higher numbers throughout the parenchyma (36, 37). In contrast, murine lymph node (LN)-resident cDC1 and cDC2 typically express CD8^+^ and CD4^+^, respectively, a fraction of LN cDC2 express CD11b, and CD103 is exclusively expressed by non-lymphoid resident (migratory) DCs (38–40). Lung resident DCs that have migrated to the MedLN are more mature, more potent T cell activators (41), and better inductors of lung-homing T cells (34) than LN-resident DCs.

In the current study, we assessed the cellular and molecular mechanisms that contribute to the induction of lung-homing CD4^+^ T cells. To reduce the experimental artifacts that are typically associated with *ex vivo* DC manipulation, we employed an immunization model comparing the intranasal (i.n.) and intramuscular (i.m.) routes, activating and loading DCs *in vivo* using various adjuvants. We demonstrate the involvement of a DC subset that is enriched in the MedLN and capable of priming lung-tropic CD4^+^ T cells. We subsequently characterize the unique surface features of this cell subset to identify some of the mechanisms at play.

## Materials and Methods

### Mice

C57BL/6J mice (BL6) and C57BL/6-Tg (TcraTcrb) 425Cbn/Crl CD45.1 (“OT-II,” OVA_323−339_ TCR transgenic) were purchased from Charles River (Ecully, France), and Rag2^−/−^ mice were a kind gift from the laboratory of Prof. Walter Reith at the University of Geneva, Switzerland. Mice were bred or housed in pathogen-free animal facilities at the University of Geneva in accordance with local guidelines. Experiments were comprised of all male cohorts at 6–8 weeks of age unless otherwise indicated, and conducted in accordance with the Geneva veterinary office, and European guidelines.

### Antibodies

The following flow cytometry detection reagents were sourced from the indicated manufacturers, Biolegend (CA, USA): anti-mouse MHC-II (I-A/I-E, clone M5.114.15.2:PE-CF594, or PerCP-Cy5.5), CD11c (clone N418:PE-Cy7), CD49a (clone HMa1:APC) CD86 (clone GL1:PerCP-Cy5.5), podoplanin (PDPN, clone 8.1.1:APC), CD11b (clone M1/70:APC-Cy7 or BV421), CD24 (clone M1/69:PE or BV421, clone SN3:PerCP eFluor710), CXCR6 (CD186, clone SA051D1:PE), CCR4 (CD194, clone 2G12:PE), CD103 (clone 2E7:PE or BV510), B220 (clone RA3-6B2:BV711), CD64 (clone X54-5/7.1:APC), CXCR3 (CD183, clone CXCR3-173:PE), CD62L (clone Mel-14:PerCP-Cy5.5), CD44 (clone IM7:APC-Cy7), CXCR4 (CD194, clone L276F12:BV421), CD45.1 (clone A20:BV650, FITC, or Pacific Blue), CD45.2 (clone 104:APC-Cy7 or PE-Cy7). Becton Dickenson (BD, NJ, USA) and BD Pharmingen (SJ, USA): CD19 (clone 1D3:BUV737), CD4 (clone RM4-5:BV786), B220 (clone RA3-6B2:PECF594), CD69 (clone H1.2F3:PE-CF594), CD49a (clone HA31/8:BV510). eBioscience (SC, USA): CD45 Ab (clone 30-F11: PECy7), CXCR5 (clone SPRCL5:PeCy7), α4β7 (LPAM1, clone DATK32:APC).

### Immunization and Injections

Mice were immunized with 40 μl of sterile PBS containing the indicated immunogen and adjuvant admixture, either by the i.n. route using a micropipette, or the i.m. route in one thigh muscle using a 30G needle and syringe. The immunogen admixtures comprised of one or more of the following as indicated in the Figure legends: endotoxin free ovalbumin (OVA) protein (Invivogen, SJ, USA); 20 μg, ovalbumin Alexa Fluor 488 conjugate (‘fluorescent OVA’, Invitrogen): 8 and 20 μg of one of the following TLR agonists (Invivogen) Poly(I:C), CpG 1826, or LPS.

For *in vivo* staining to distinguish total leukocytes in the lung vasculature compared to the lung parenchyma, described elsewhere in detail (42), anti-CD45 Ab (clone 30-F11: PECy7, eBioscience) was diluted 1/50 in sterile PBS (Sigma, SL, USA) and injected i.v. into anesthetized mice. Mice were sacrificed exactly 2 min after, lungs were removed immediately, and processed as described below without prior perfusion. When indicated, detection of CD45.1^+^ OT-II cells during the *ex vivo* surface staining procedure was performed using anti-CD45.1 Ab (clone A20: Pacific Blue, Biolegend).

### Tissue Harvesting and Cell Preparation

After euthanasia via CO_2_ inhalation, the LNs or spleens were removed, dissociated using a mortar and pestle, digested with 1 μg ml ^−1^ Liberase TL (Roche, Basel, Switzerland) and 1 mg ml ^−1^ DNase I (Sigma-Aldrich) in serum free media for 30 min at 37°C. Single-cell suspensions were filtered, counted, and washed in DC media (RPMI 1640 media (Invitrogen) supplemented with 10% heat-inactivated (HI) FCS (Pan Biotech, Germany), 25 mM HEPES, 50 U ml ^−1^ penicillin (Gibco, MA, USA), 50 μg ml ^−1^ streptomycin (Gibco), 50 μM 2-mercaptoethanol, sodium pyruvate 1 mM (Sigma), non-essential amino acids at the recommended dilution (Gibco) and 2 mM EDTA (Sigma), before staining for cytometry. When necessary, red blood cell lysis was performed using ACK buffer (BD) for 2 min at room temperature.

Lung tissue was minced and then digested using 5 ml of serum-free RPMI containing 350 enzyme activity units of Collagenase IV (Worthington Biochem, NJ, USA) and 5 mg of DNase I, and incubated for 30 min at 37°C before being passed through a 20 μm filter, washed, counted, subjected to RBC lysis, and then plated similarly to other tissues.

### Cell Enrichment or Depletion

OT-II cells used for *in vitro* co-culture and *in vivo* transfer experiments were enriched from the spleens of naïve OT-II mice using a negative selection “naïve CD4^+^ T cell enrichment kit” (Miltenyi, Germany). Purity was consistently between 88 and 96% CD4^+^ cells, with >99% of these cells being CD44^−^, as confirmed by flow cytometry.

CD3^+^, and or CD19^+^ cells were sometimes depleted from LN single cell suspensions using positive selection magnetic bead-based cell enrichments kits MACS kits (Miltenyi) as described in the manufacturer protocol.

### Flow Cytometry Staining, Acquisition, and Cell Sorting

One to three million cells were added to 96-well V-bottom plates (ThermoScientific, MA, USA) and washed in RT PBS, then incubated at RT for 10 min in one of the following viability dyes diluted 1/1,000 in RT PBS; LIVE/DEAD Fixable Blue or Aqua (Invitrogen), or Zombie Yellow Fixable Viability Kit (Biolegend). Cold conditions and reagents (1–4°C) were used for the following steps. Wells were completed with FACS buffer [“FB,” comprising of PBS, with 2% FCS, and 0.05% azide (Sigma)] containing 50 μg ml ^−1^ Fc block (mAb 2.4G2), centrifuged, re-suspended in cocktails of fluorescently-tagged Abs or isotype-control Abs for 20 min, before washing in FB, fixed for 20 min using a FoxP3 fixation kit (eBioscience) as per the manufacturers' recommendations. Cells were then washed in FB and acquired using an HTS plate reader and LSRFortessa cytometer (BD). To more accurately enumerate cell numbers, samples were spiked with set amounts of 10 μm Coulter CC latex beads (Beckman Coulter) during the staining procedure. To determine cell size, the Tali™ image-based cytometer (Beckman Coulter) was used to acquire between 200 and 375 events from (i) sorted DC subsets that had been rested overnight in complete media, at 37°C with 5% CO_2_, (ii) MACS-purified naïve OT-II cells, or (iii) 10 μm Coulter CC latex beads. Dead cells were excluded using a viability dye and doublets were removed during the analysis.

Screening of DC surface proteins made use of the Biolegend LEGENDScreen® Lyophilized Ab Array containing 255 PE-conjugated Abs specific for mouse cell markers, including 11 isotype-matched control PE-conjugated Abs (see Figure 4D and Table S1), performed as per the manufacturers' instructions.

Flow cytometric sorting of DCs was performed at the University of Geneva Flow Cytometry Platform, using a MoFlow Astrios flow cytometer. DC suspensions were optionally depleted of CD19^+^ and CD3^+^ lymphocytes as described above to increase the sorting rate. Cell suspensions were stained with fluorescent Abs that were specific for leukocyte lineage markers, then diluted in azide-free FACS buffer and sorted into tubes containing complete media using the following gates; singlets, live, SSC vs. FSC for DC morphology, exclusion of autofluorescent and CD64^hi^ cells [macrophages and inflammatory monocytes (43)], exclusion of NK1.1^+^ (NK cells), B220^+^ (B cells and plasmacytoid DCs), and CD3^+^ cells, followed by positive selection of the desired DC population using markers including MHC-II, CD11c, CD103, CD11b, and CD24. Sorted DC subsets were routinely >99% pure, and were washed before being used immediately in T cell co-culture assays.

### Flow Cytometry Analysis

The net geometric mean fluorescence intensity (gMFI) in the LEGENDScreen® analysis was calculated by subtracting the background PE fluorescence, which was obtained using either a PE-conjugated isotype control Ab or fluorescence-minus-one staining control (full Ab panel in the absence of a PE-conjugated Ab). The ratio of gMFI was calculated by dividing the net PE gMFI of OVA^+^ CD11b^+^ MedLN DCs by the net PE gMFI of OVA^+^ CD11b^+^ ILN DCs.

Principal Component Analysis was performed with 500 randomly selected Ag^+^ CD11b^+^ conventional DC events, from each of two draining LN sites immunized mice, using FCS Express 6 software.

*t*-SNE analysis was performed using Flowjo software (Tree Star, USA). Briefly, a total of 2,500 randomly selected Ag^+^ CD11b^+^ conventional DC events, from the draining ILN and MedLN of immunized mice were concatenated. The t-SNE dimensionality reduction was performed using these 5,000 concatenated cells and the following parameters: theta = 0.5, eta = 200, perplexity = 20, and iterations = 1,000, and the expression of CD86, CD24, PDPN, and GL7. These parameters were defined empirically and selected because they resulted in adequate separation between cell sub-phenotypes on the bivariate *t*-SNE “x” vs. *t*-SNE “y” plot. Nine gates were drawn on the bivariate t-SNE plot to further analyze marker expression within the major sub-phenotypes using histograms, including in comparison to conventionally gated non-DC control subsets; B (CD19^+^CD11c^−^CD4^−^) or CD4^+^ lymphocytes (CD4^+^CD3^+^MHC-II^−^).

### *In vitro* DC/T Cell Co-cultures

MACS-purified OT-II cells and cytometry-sorted DCs were plated in 96-well U-bottom plates (ThermoScientific, MA, USA) in 100 μl of DC culture media (EDTA-free) at a ratio of 20 to 1, comprising of 5,000 DCs and 100,000 OT-II cells per well, in the absence of exogenous cytokines or antigens. The co-culture was maintained in a humidified incubator for 1–3 days at 37°C with 5% CO_2_. Co-cultures were performed using DCs and OT-II cells from gender and age-matched mice.

Blockade of DC surface markers during co-culture was achieved by incubating 5,000 sorted DCs in 10 μg ml ^−1^ (saturating concentrations) of one of the following purified mAbs: rat IgG2b anti-CD24 (clone M1/69), rat IgM anti-GL7 (clone GL7), Syrian hamster IgG anti-PDPN (clone 8.1.1), rat IgG2a anti-CD86 (clone GL1), or matched isotype control mAbs, for 20 min on ice, before washing the cells, co-culturing with 100,000 OT-II cells and staining for flow cytometry as described above.

### *In vivo* T Cell Trafficking

MACS-purified naïve OT-II cells (200,000 per mouse) were transferred via the i.v. route into naïve age and gender-matched BL6 recipients before immunization as described above. Ten days later, *in vivo* staining of vascular/parenchymal lung leukocytes was performed followed by *ex vivo* staining of OT-II cells and characterization of the T cell response by flow cytometry, all as described above.

To compare the imprinting capacity of DCs in the absence of other cells and on a “per-cell” basis, a competitive adoptive-transfer T cell assay was performed using naïve OT-II cells that were activated for 3 days using *in vitro* co-culture with sorted Ag^+^ CD11b^+^ DCs from immunized mice, as described above. As an additional comparison, naïve OT-II were activated using *A*g^−^ CD11b^+^ DCs from the dLNs of immunized mice, which were pulsed with 0.1 μg ml ^−1^ OT-II peptide (OVA_323−339_, Invivogen) in complete media for 30 min, and washed twice, before co-culture. After 3 days of activation by co-culture with OVA^+^ DCs or OVA^−^ Ag-pulsed DCs, a live cell count was performed, and OT-II cells were adjusted to 5 million ml ^−1^ in PBS containing 0.1% bovine serum albumin (BSA, Sigma Aldrich). Cells from different co-cultures were stained with either high (20 μM) or low (0.5 μM) concentrations of CFSE (Molecular Probes) in PBS containing 0.1% BSA, for 10 min at 37°C, before quenching with an equal volume of cold HI FCS. Live CFSE^high^ and CFSE^low^ stained cells were mixed at a 1:1 ratio. One hundred thousand cells from each DC site co-culture (200,000 per mouse) were injected i.v. using a 30G syringe into the tail vail of anesthetized recipient Rag2^−/−^ mice, which were matched in terms of age and gender to the co-culture cell donor mice. After 18 h, *in vivo* and *ex vivo* staining was performed to enumerate and characterize OT-II cells in the lung parenchyma vs. lung circulation, spleen and dLNs, as described above. The tissue homing index is the number of CD44^+^ OT-II cells in a given tissue compartment that were originally primed by MedLN DCs, divided by the number of CD44^+^ OT-II cells in the same tissue originally primed by ILN DCs. This index was normalized because different proportions of CD44^+^ cells were present in the 1:1 mixture (based on total live cells) of MedLN and ILN DC-primed co-cultures prior to transfer. Normalization involved division of each homing index value by the % CD44^+^ of total OT-II cells from each co-culture type in the 1:1 mixture, thereby reducing the homing index of OVA^+^ MedLN DCs and increasing the homing index generated by OVA^+^ ILN DCs.

### Statistical Analysis

Statistical significance was calculated with GraphPad Prism (CA, USA) using either an ANOVA with a Tukey post-test, two-tailed unpaired *t*-tests with Holm-Sidak multiple comparison correction and alpha of 0.05, two-tailed ratio-paired *t*-test with 95% CI, single sample *t*-tests with 0.05 alpha significance and expected values of either 1 (for ratio comparisons) or 100 (for percentage response comparisons), or two-way ANOVA with Tukey post-tests, depending on the number of groups and type of comparison, as indicated in the legends. Error bars in graphs show the S.D. when comparing groups of individual mouse data points, or alternatively the S.E.M. when data points represent pooled data from independent experiments.

## Results

### Higher Proportions of Ag^+^ dLN cDC1 and cDC2 and Lung Homing T Cell Responses Are Elicited by Intranasal Compared to Intramuscular Immunization

**Figure 1:**
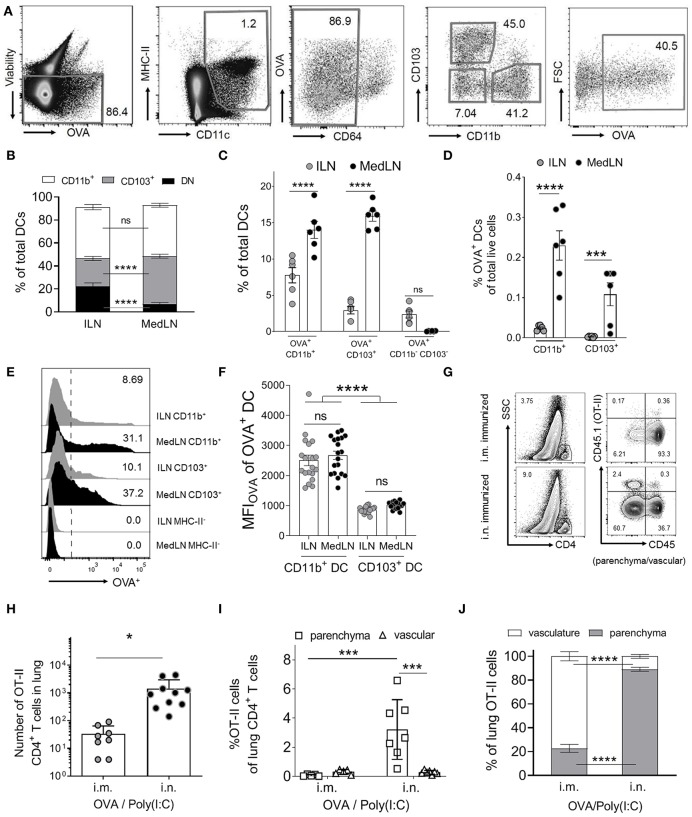
Intranasal immunization results in higher percentages of specific Ag^+^ DC subsets and increases lung-trafficking of CD4^+^ T cells. **(A–F)** Draining LN (dLN) suspensions from mice immunized i.n. or i.m. with fluorescent Ag (OVA) and Poly(I:C) 24 h earlier were stained with specific Abs prior to flow cytometric analyses. **(A)** Representative cytometry plots of the gating strategy for conventional DCs, based on (43). DCs were defined as viable (non-autofluorescent, B220^−^, singlets; not shown), MHC-II^+^, CD11c^+^, CD64^low/int^. DC subsets were based on CD11b, CD103, and Ag (OVA) expression. **(B)** Shows the percentage of DC subsets from total conventional DCs, **(C)** the percentage of OVA^+^ cells within DC subsets and **(D)** the percentage of OVA^+^ DC subsets as a percentage of total live dLN cells. **(B–D)** Show 6 pooled experiments with 6–12 mice per route per experiment. **(E)** Representative histograms of the OVA^+^ signal in selected DC subsets and an MHC-II^−^ control cell subset. **(F)** Shows the MFI_OVA_ of the indicated DC subsets; 19 experiments pooled, 3–12 mice per route per experiment. **(G–J)** Purified naïve CD4^+^ OVA-specific (OT-II) T cells were transferred into naive CD45.1^−^ BL6 mice prior to i.m. or i.n. immunization with OVA/Poly(I:C). Ten days later, *in vivo* staining of vascular leukocytes was performed immediately before removing the lungs to stain CD45.1^+^ OT-II cells. **(G)** Shows the gating strategy for representative mice from one experiment, to identify OT-II cells in the lung parenchyma (CD45^−^) or vasculature (CD45^+^), after gating SSC^low^ CD4^+^ T cells (singlet, viability, and CD3^+^ gates not shown). OT-II cells in the indicated lung compartments are shown as **(H)** total numbers or **(I)** as percentages of total CD4^+^ cells or **(J)** total OT-II cells in the lung, with individual mice (*N* = 8–10), pooled from two or more independent experiments. Statistical significance was calculated using an ANOVA when comparing three or more groups, or a *t*-test for two groups, with SEM bars shown for pooled averages of multiple experiments, or SD bars for pooled individual mouse data, as detailed in the Materials and Methods. *P* > 0.05; “ns,” not significant; ^*^P < 0.05; ^***^P < 0.001; ^****^P < 0.0001.

We initially characterized the general DC response in dLNs 24 h post i.n. or i.m. immunization with fluorescent ovalbumin protein antigen (OVA Ag) and Poly(I:C). Based on CD11b and CD103 expression, we detected three distinct conventional DC subsets in the draining MedLN and ILN (Figures 1A–C): CD11b^+^, CD103^+^, and double negative (DN) CD11b^−^ CD103^−^ DCs. CD11b^+^ DCs comprised of similar proportions of total DCs in both dLN sites, whereas the CD103^+^ subset represented higher, and the DN subset represented lower frequencies in the MedLN compared to the ILN (Figure 1B) of immunized mice. This was in contrast to naïve mice, where all three major DC subsets were present in both LN sites at equal proportions at steady state (Figure S1A). Of the tested time-points, the percentage of Ag^+^ cells was highest on day 1 and had waned by day 2–3 depending on the subset and site (Figure S1B). There were higher percentages of Ag^+^ CD11b^+^ and CD103^+^ DCs in the MedLN compared to the ILN (Figure 1C), however because of the differing total numbers of DC subsets per ILN and MedLN, the absolute number of OVA^+^ CD11b^+^ DCs was similar between sites, and the absolute number of OVA^+^ CD103^+^ DCs was higher in the MedLN (Figures S1C,D). OVA^+^ DCs comprised of more than 2.5% of all three DC subsets, except for the DN DC subset in the MedLN (Figure 1C). Given the low number per MedLN (Figures S1C,D) and very low percentage of Ag^+^ DN MedLN DCs (Figure 1C), this subset was not investigated further. Notably, the overall density of Ag^+^ DCs was higher in the MedLN compared to the ILN (Figure 1D). Additionally, the mean fluorescence intensity (MFI) of Ag in Ag^+^ cells appeared higher in CD11b^+^ compared to CD103^+^ DCs (Figures 1E,F), regardless of which LN site was compared. There were no differences in Ag MFI between the same DC subsets from ILN and MedLN (CD11b^+^ DC; CD103^+^ DC; Figure 1F).

Next, we investigated whether i.n. immunization elicits an enhanced lung-trafficking CD4^+^ T cell response. Naïve (CD44^−^) OVA-specific CD4^+^ T cells (OT-II) were adoptively transferred into recipient naïve BL6 mice, which were then immunized i.n. or i.m. with OVA/Poly(I:C). Post-transfer of OT-II cells, and immediately prior to sacrifice, *in vivo* intravenous staining (42) was used to discriminate parenchymal and vascular lung T cell localization (Figure 1G). Significantly higher numbers of OT-II cells were detected in the lung after i.n. immunization compared to after i.m. immunization (Figure 1H). Airway priming of T cells also resulted in an enrichment of OT-II cells in the lung parenchyma as a percentage of total CD4^+^ T cells (Figure 1I), or as a percentage of OVA-specific T cells (Figure 1J). Globally, these results confirmed that i.n. immunization increases the homing of CD4^+^ T cells into the lung interstitium compared to i.m. immunization.

### Increased Priming and Induction of Lung-Homing Markers on CD4^+^ T Cells by *in vivo*-Activated MedLN DCs Compared to ILN DCs

**Figure 2:**
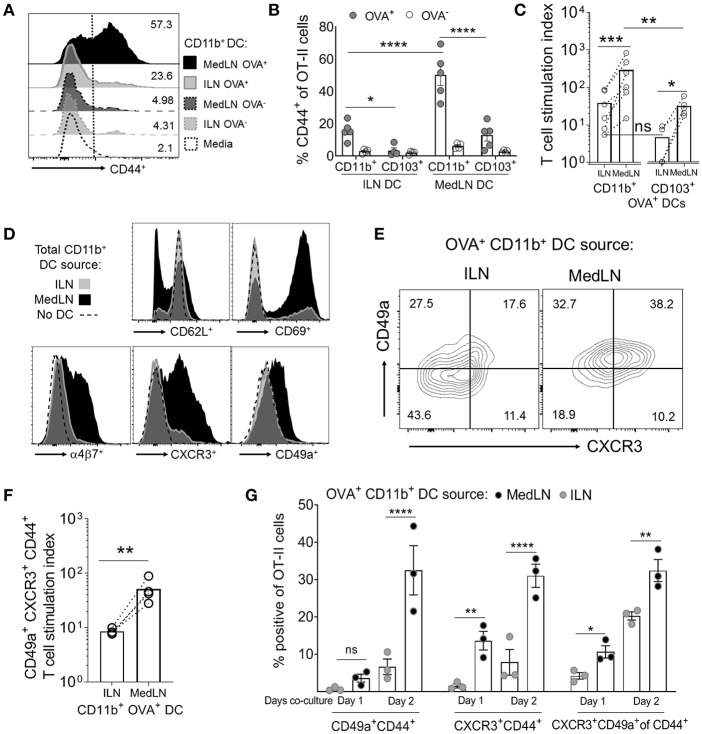
*In vivo*-activated DCs in the lung- and muscle-draining LNs differentially prime CD4^+^ T cells in terms of speed, magnitude, and induction of lung homing molecules. **(A–G)** Mice were immunized by the i.n. or i.m. route with fluorescent OVA and Poly(I:C). After 24 h, the indicated DC subsets (5,000 cells per subset) were sorted from dLN suspensions and co-cultured with 100,000 naive OT-II CD4^+^ T cells for 1–3 days in the absence of exogenous cytokines or antigen. **(A)** Representative flow cytometry histograms of CD44 expression of gated OT-II cells after 3 days of DC co-culture. **(B)** The percentage of CD44^+^ OT-II cells after 3 days of co-culture with either OVA^+^ or OVA^−^ CD11b^+^ or CD103^+^ DC subsets from the draining ILN or MedLN of immunized mice. **(C)** OVA^+^ DC subset priming of OT-II cells, expressed as a stimulation index that represents the fold-change in OT-II numbers per well, compared to the media control. **(D)** Representative histograms of OT-II CD4^+^ T cell phenotype after 3 days of DC co-culture. **(E–G)** OVA^+^ CD11b^+^ DCs were sorted from the dLNs of i.m. or i.n. immunized mice and co-cultured with naïve OT-II cells for 3 days or as indicated. **(E)** Representative flow cytometry contour plots of CD49a and CXCR3 expression of CD44^+^ CD4^+^ T cells after DC co-culture with OT-II cells on day 2 of *in vitro* activation. **(F)** OVA^+^ CD11b^+^ DC subset induction of lung homing molecules CXCR3 and CD49a on OT-II cells, expressed as a stimulation index, which represents the fold-change in numbers per well, compared to the media control OT-II cell culture. **(G)** The percentage of gated OT-II cells expressing the indicated lung-homing molecules after 1 or 2 days of DC co-culture. In **(B,C,F,G)**, points represent the mean response from individual experiments with SEM bars. An ANOVA was used in **(B,G)**, and a two-tailed ratio *t*-test was used in **(C,F)**, as described in the Materials and Methods. *P* > 0.05; “ns,” not significant; ^*^P < 0.05; ^**^P < 0.01; ^***^P < 0.001; ^****^P < 0.0001.

Because the two routes of immunization elicited different lung-homing T cell responses, we then directly compared the ability of MedLN and ILN DCs to prime naïve T cells. CD11b^+^ and CD103^+^ DC subsets harvested from the dLNs of OVA/Poly(I:C)-immunized mice were cytometry-sorted and co-cultured with purified OT-II cells in the absence of exogenous cytokines or antigens. T cell activation, as measured by CD44 expression (44) (Figure 2A) of gated OT-II cells (Figure S2A) was detected at early time-points (day 1) and continued increasing at day 3 (Figure S2A and Figure 2B). The most potent activating DCs were Ag^+^ MedLN CD11b^+^ DCs, which on average elicited 55% CD44^+^ OT-II cells (Figure 2B) and had a T cell stimulation index of 260 times that of the media-only stimulated OT-II cells (Figure 2C). This level of T cell activation was significantly higher compared to Ag^+^ CD11b^+^ DCs from the ILN (Figure 2B). All Ag^−^ DC subsets elicited minimal (1–8%) T cell activation (Figure 2B), demonstrating that Ag was required to elicit robust T cell activation. Antigen-positive CD103^+^ MedLN DCs primed significantly higher numbers (Figure 2C) of T cells compared to their equivalent counterparts from the ILN. However, OT-II priming mediated by CD103^+^ (cDC1) DCs was on average 10.15 times lower than priming by CD11b^+^ DCs, confirming the observations that show specialization of cDC1 and cDC2 subsets in priming CD8^+^ and CD4^+^ T cell responses, respectively (45–47). We therefore focused on CD11b^+^ DCs to further evaluate CD4^+^ T cell priming by MedLN vs. ILN DCs.

We assayed for changes in the expression of 9 airway-associated chemokine receptors and integrins, as well as cell activation or lineage markers on the surface of OT-II CD4^+^ T cells after co-culture with total CD11b^+^ DCs from the MedLN or ILN of immunized mice (Figure 2D and Figure S2B). This initial screening demonstrated that MedLN-derived DCs induced high percentages of T cells that expressed CD69, α4β7, CXCR3, and CD49a, and reduced the expression of CD62L more visibly than ILN DCs (Figure 2D). Furthermore, Ag^+^ MedLN DCs induced higher percentages (Figure 2E) and numbers (Figure 2F) of lung homing CXCR3^+^ CD49a^+^ OT-II cells, including at multiple time-points of the co-culture (Figure 2G; day 1, and Figure S2C; day 3). Ag^+^ MedLN-derived CD11b^+^ DCs could selectively enhance the induction of lung homing OT-II cells (Figure 2G; far right graph), as determined by first gating on CD44^+^ cells to normalize for the variation between the overall T cell priming capacity of OVA^+^ ILN and MedLN DCs (Figure 2A). We were unable to detect CCR4, previously described in T cell co-cultures with airway-DCs (34) or other selected molecules that have been implicated with lung-homing (CXCR4, CXCR5, CXCR6; Figure S2B) (6, 34, 48), including in co-cultures of up to 5 days (data not shown). Increased percentages of endogenous (polyclonal) CXCR3^+^ CD49a^+^ double positive cells were also observed at higher frequencies directly *ex vivo* in the CD44^+^ CD4^+^ T cell compartment of the MedLNs of i.n.-immunized BL6 mice compared to the ILN of i.m. immunized mice (Figures S2D,E).

### MedLN CD11b^+^ DCs Induce CXCR3 and CD49a Expression on CD4^+^ T Cells That Home to the Lung Parenchyma

**Figure 3:**
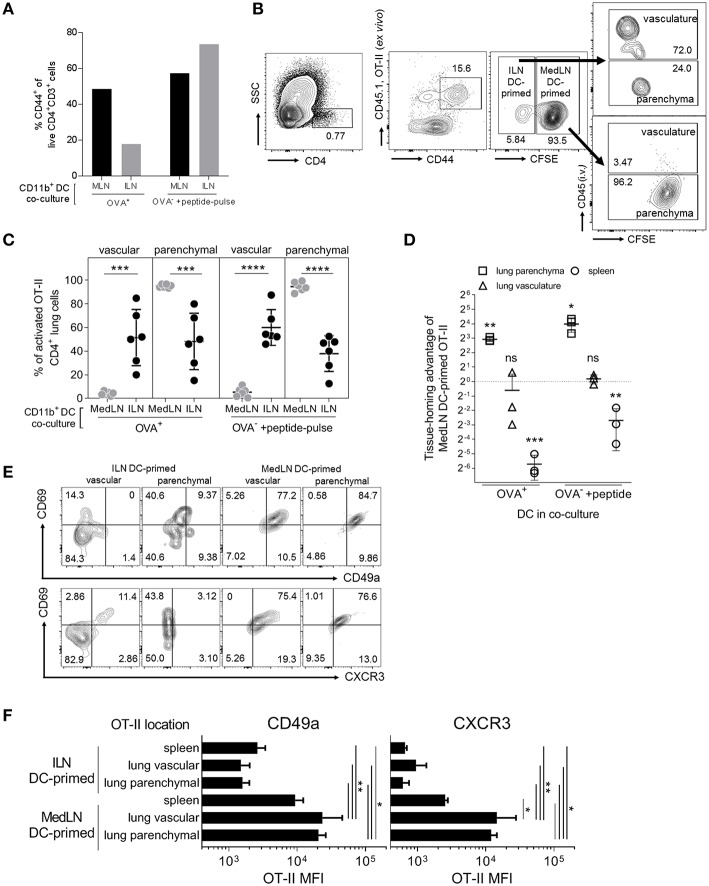
*In vivo* lung parenchyma-homing advantage of OT-II cells primed by MedLN DCs. OT-II cells were co-cultured for 3 days with OVA^+^ or OT-II peptide-pulsed OVA^−^ CD11b^+^ DCs from the draining ILN or MedLN of immunized mice. **(A)** Co-cultured OT-II cells were stained for flow cytometric analysis to determine CD44 expression, one representative experiment shown. Cells from MedLN or ILN DC co-cultures were labeled with high or low concentrations of CFSE, respectively, and mixed at a 1:1 ratio of live cells before i.v. injection into T cell deficient Rag2^−/−^ mice (*n* = 6 per DC co-culture type). The following day, mice were injected i.v. with labeled anti-CD45 Ab prior to sacrifice to distinguish parenchymal vs. vascular lung leukocytes, and organs were harvested for flow cytometric analysis. **(B)** Flow cytometry contour plot gating strategy using 6 concatenated samples to distinguish the lung compartment and cell-culture origin (ILN or MedLN DC primed) of the transferred OT-II cells recovered *ex vivo*. **(C)** The percentage of activated OT-II from DC co-cultures detected in different lung compartments. **(D)** The tissue homing index, expressed as a ratio of the number of MedLN to ILN DC-primed (CD44^+^) OT-II cells detected in different tissues after normalization, see Materials and Methods. **(C,D)** Data represents individual mice (*n* = 3–6) from one of two independent experiments. **(E,F)** Phenotype of CD44^+^ OT-II cells detected in recipient mice lung compartments, shown as **(E)** concatenated flow cytometry contour plots, or **(F)** the expression intensity of the indicated lung homing markers, from six mice representing one of two independent experiments. Statistical analysis comprised of a one way ANOVA in **(C,F)**, single sample *t*-test in **(D)**, and SD error bars, as detailed in the Materials and Methods. *P* > 0.05; “ns,” not significant; ^*^P < 0.05; ^**^P < 0.01; ^***^P < 0.001; ^****^P < 0.0001.

DC-induction of *in vivo* T cell homing capacity was investigated by using sorted CD11b^+^ DCs to prime OT-II cells, which were then transferred into naïve lymphocyte-deficient (RAG2^−/−^) recipient mice. As the overall level of T cell activation by ILN DCs was lower, we additionally used OT-II peptide pulsing of Ag^−^ DCs as a comparison to the standard *in-vivo* loaded Ag^+^ DC co-culture, which approximately equalized *in vitro* T cell activation by DCs from either the ILN or MedLN (Figure 3A). Prior to transfer, the T cells from MedLN and ILN DC co-cultures were differentially stained to allow discrimination of their co-culture origin *in vivo*. Post-transfer, and immediately prior to sacrifice, additional staining was performed to determine the localization of OT-II cells within the lung (Figure 3B). MedLN-derived DCs imprinted cell-homing capabilities that resulted in greater percentages (Figure 3C; *P* < 0.001 for all MedLN and ILN comparisons) and numbers (Figure 3D; OVA^+^ DCs) of activated OT-II in the lung parenchyma *in vivo* (Figure 3D; OVA-pulsed DCs), even after normalizing for the difference in CD44^+^ cells in the 1:1 mixture of live co-cultured OT-II cells (see Figure 3A) that were then adoptively transferred. In contrast, ILN DC-primed OT-II showed a strong homing preference for the spleen, equivalent to over 10-fold in terms of absolute numbers (Figure 3D). Approximately the same number of MedLN and ILN DC primed (CD44^+^) OT-II cells were detected in the lung vasculature post-transfer (Figure 3D; *p* > 0.05). A relatively high number of MedLN-DC primed OT-II cells were unaccounted for, suggesting either cell death, or homing to other sites, including the mucosa. The latter is plausible considering that the gut-homing molecule α4β7 was induced by intranasal-route DCs in our study (Figure 2D) and previously by other experimental airway-delivered vaccines (49–51), in addition to other known redundancies in tissue-trafficking molecule specificity (52, 53). Too few of the *in vitro* activated OT-II cells were detected in the LNs post-transfer to perform accurate quantitative or qualitative analyses.

The lung parenchyma-homing advantage mediated by MedLN CD11b^+^ DCs was further supported by an analysis of the phenotype of activated (CD44^+^) OT-II cells post *in vivo* transfer (Figures 3E,F; Figure S3A). High percentages of MedLN DC-primed OT-II cells co-expressed the activation and tissue retention marker CD69 with either CD49a or CXCR3, whereas CD69 was expressed mainly in the absence of these lung-homing markers in ILN DC-primed OT-II cells (Figure 3E). Over 75% of all OT-II cells activated by MedLN DC, which were isolated from the lung parenchyma expressed CD69 (54, 55) in addition to either CD49a or CXCR3, whereas <10% of ILN DC-induced parenchymal OT-II cells were double-positive for combinations of these markers (Figure 3E). MFI analysis demonstrated that both CD49a and CXCR3 were expressed on average at higher levels in MedLN DC-primed OT-II cells found in both the lung interstitium and parenchyma compared to OT-II primed by ILN DCs (Figure 3F), and additionally in the vasculature for CXCR4 (Figure S3B). CXCR3 expression of MedLN DC-activated OT-II cells was significantly higher in T cells found in the lung compared to the spleen, with a similar trend for CD49a (Figure 3F). This suggests that CXCR3 may be a more specific lung-homing molecule or is upregulated to a higher degree compared to CD49a in our model.

### Identification of Site-Specific Differences in dLN CD11b^+^ DC Surface Markers, Including Co-expression of High Levels of Multiple Co-stimulatory and Activation Markers on MedLN DCs

**Figure 4:**
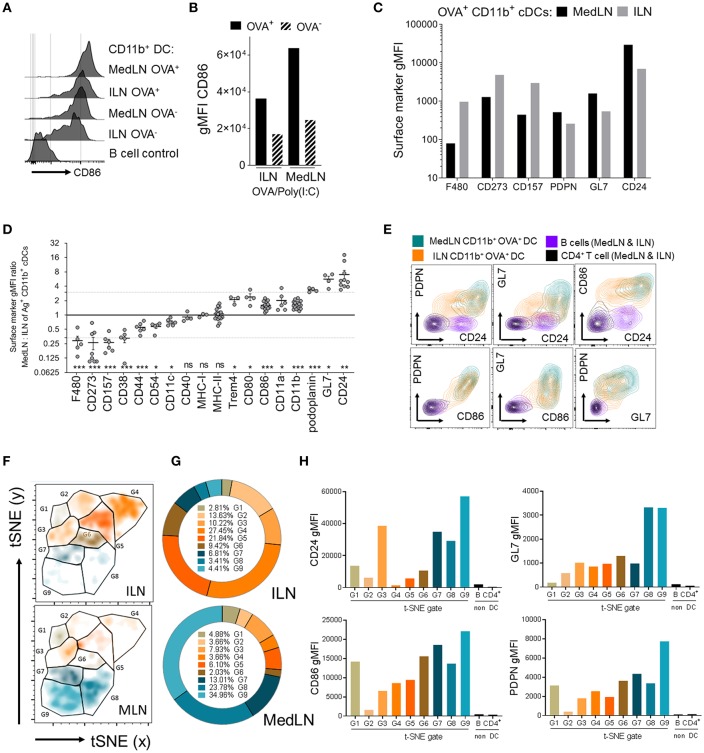
Phenotypic differences between MedLN and ILN CD11b^+^ DCs. Draining LNs of male or female mice, after i.n. or i.m. immunization 24 h earlier with fluorescent OVA and Poly(I:C), stained for flow cytometric analysis. **(A)** Shows histograms of CD86 expression, **(B)** shows CD86 MFI of OVA^+^ or OVA^−^ CD11b^+^ DCs, and **(C)** shows the MFI of various markers in CD11b^+^ DCs from the indicated LNs. **(A–C)** Show data from one of three similar experiments **(D)** shows the ratio of gMFI for the indicated markers from MedLN to ILN Ag^+^ CD11b^+^ DCs, calculated after normalizing each sample with isotype or “fluorescence minus one” Ab staining controls. The dotted line indicates an arbitrary 3-fold higher or lower ratios of expression, individual points represent experimental repeats, pooling LNs from 3 to 12 mice per immunization route, with *p*-values calculated using a single-sample *t*-test (0.05 alpha, expected MFI ratio of 1). **(E)** Co-expression of the indicated surface markers in the indicated cell subsets from one of three similar experiments. **(F)** Shows a t-SNE analysis of an equal number of OVA^+^ CD11b^+^ cDCs from the dLNs of i.n. or i.m. immunized mice as described in the Materials and Methods. *t*-SNE data was used to generate **(G)** pie graphs showing the percentage of cells in each *t*-SNE gate, relative to total OVA^+^ CD11b^+^ cDCs from the same LN, and **(H)** column graphs show the cell surface gMFI of the indicated markers in each of the *t*-SNE gates using combined ILN and MedLN data. *t*-SNE data is representative of three independent experiments. *P* > 0.05; “ns,” not significant; ^*^P < 0.05; ^**^P < 0.01; ^***^P < 0.001.

Data showing that MedLN DCs were superior to ILN DCs at activating CXCR3^+^ CD49a^+^ CD4^+^ T cells prompted a detailed analysis of DC phenotype to gain insight about the imprinting mechanisms at play during T cell priming. We initially investigated the expression of CD86, a well-characterized T cell co-stimulatory molecule which is often used to indicate stimulatory capacity or the activation status of antigen-presenting cells. At 24 h post-immunization, CD86 was found at higher levels on the surface of Ag^+^ CD11b^+^ DCs from the MedLN compared to the ILN (Figures 4A,B). We excluded the possibility that MFI differences in surface molecule expression were due to cell size using image-based cytometry (Figure S4A), or due to kinetics by harvesting the dLNs at later time points post-immunization (Figures S4B,C). Although baseline levels of CD86 were slightly higher in ILN-derived DCs (Figures S4B,C; naïve mice), much higher CD86 expression was consistently detected in Ag^+^ MedLN-derived DCs at day 1 post-immunization, and required several days to return to baseline levels (Figures S4B,C).

Recognizing that the expression level of a key co-stimulatory molecule was affected by the anatomical location of DCs, we then compared the expression of 254 additional leukocyte markers on the surface of Ag^+^ CD11b^+^ DCs from the ILN and MedLN at day 1 post-immunization. Several markers were expressed with a >3-fold difference on the surface of Ag^+^ CD11b^+^ DCs in the draining MedLNs vs. ILNs of OVA/Poly(I:C)-immunized mice. These included F4/80, CD273 (PD-L2), CD157, podoplanin (PDPN), GL7, and CD24 (Figure 4C). This result was reproduced upon immunization with adjuvants targeting distinct TLRs, namely CpG 1826 (TLR9) and LPS (TLR4) (Figure S4D), which suggests that the molecular surface signature of DCs from different sites is a generalized phenomenon after adjuvanted OVA immunization. The majority of surface markers were not differentially expressed on the surface of ILN and MedLN DCs (Table S1), including co-stimulatory and antigen presenting molecules, such as CD40, MHC-I, MHC-II (Figure 4D), and TGF-β (Figure S4E)—which has previously been reported to mediate imprinting of lung-homing CD103^+^ CD8^+^ T cells by CD1c^+^ human DCs (24). A few markers were statistically different between MedLN and ILN Ag^+^ DCs, however, with a lower magnitude of difference between 1.5- and 3-fold (Figure 4D). The majority of differentially expressed markers between dLN sites are well-characterized for their involvement in co-stimulation (CD80, CD86, “heat stable antigen” CD24) or co-inhibition (F4/80, CD273, CD80) of T cells, adhesion and immunological synapses (CD44, CD54/ICAM-1, CD11a, CD11b, CD11c), or are currently used as leukocyte activation markers (GL7, CD44, PDPN); Table 1 (31, 44, 56–60). Interestingly, differential expression of many of these markers was detected between MedLN and ILN DCs even prior to immunization (Figure S4B).

**Table 1:** Summary of differentially-expressed surface markers between MedLN and ILN Ag^+^ CD11b^+^ DCs.

**DC surface molecule(s)**	**LN with increased expression**	**Molecule function**
CD24 (HSA)	MedLN	T cell co-stimulation and cell migration
CD80, CD86	MedLN	T cell co-stimulation or co-inhibtion
CD11a, CD11b	MedLN	Adhesion
PDPN (podoplanin)	MedLN	Lymphatics/LN trafficking, IFN response
F4/80	ILN	Adhesion, Treg induction
CD273 (PD-L2)	ILN	T cell co-inhibitory molecule
CD38, CD157	ILN	Calcium and NAD regulation
CD11c, CD44, CD54 (ICAM-1)	ILN	Adhesion, migration, immunological synapse

*MedLN, mediastinal lymph node; ILN, inguinal lymph node; NAD, nicotinamide adenine dinucleotide; IFN, interferon; Treg, regulatory T cell*.

The three markers found at more than 3-fold higher levels on MedLN DCs (CD24, HSA; GL7; and PDPN), were investigated in more detail. By simultaneously evaluating their surface expression, in addition to CD86, we aimed to evaluate whether these markers were co-expressed on the same cell, or alternatively on mutually exclusive cell subsets. Bivariate dot plots allowed the investigation of pairs of markers, which demonstrated a high level of co-expression at the single cell level (Figure 4E) and highlighted the existence of some unique cell distributions, for example MedLN DCs that co-expressed very high levels of both GL7 and CD24. To further stratify the CD11b^+^ DC compartment based on the more highly-expressed MedLN DC surface markers, we employed the t-distributed stochastic neighbor embedding (t-SNE) dimensionality reduction algorithm. Visualization of 4-dimensional data on a 2D t-SNE plot assisted in the identification of a DC subphenotype that simultaneously expressed high levels of CD24, CD86, GL7, and PDPN (Figures 4F–H). This cell sub-phenotype (labeled G9, Figures 4F–H), represented over 34% of Ag^+^ CD11b^+^ DCs in the MedLN, and under 5% of Ag^+^ CD11b^+^ DCs in the ILN (Figure 4G). The MFI analysis of each of the 9 gated sub-phenotypes (G1–G9, histograms in Figure S4F) revealed that the most abundant Ag^+^ CD11b^+^ DCs in the ILN (G4 and G5) expressed low levels of CD24 and intermediate levels of CD86 co-stimulatory molecules, and less abundant sub-phenotypes (~10% of total Ag^+^ cells, e.g., G3 and G6) did not simultaneously express moderate or high levels of more than one type of co-stimulatory (CD86 or CD24) or activation marker (PDPN or GL7).

### Lung-Homing CD4^+^ T Cells Are Efficiently Induced by Ag^+^ CD24^+^ CD11b^+^ DCs, Which Are Enriched in the MedLN Compared to the ILN

**Figure 5:**
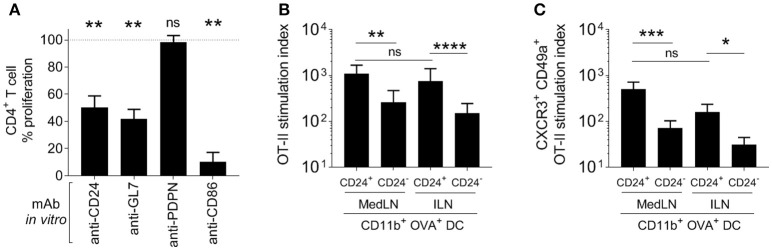
Role of specific DC surface molecules and subsets in the activation of T cells and the induction of lung-trafficking molecules. **(A)** Draining MedLN OVA^+^ CD11b^+^ DCs from mice immunized 24 h earlier, were incubated with isotype control Abs or the indicated blocking mAbs before co-culture and evaluation of % CD44^+^ OT-II cells. Values are relative to the isotype control reference (100%) for 3–5 independent experiments, showing SEM error bars and compared using a one sample *t*-test with expected value of 100%. In **(B,C)**, 5000 OVA^+^ CD24^+^ or CD24^−^ DCs were isolated from the dLNs of i.n. or i.m.-immunized mice and co-cultured with 100,000 naïve OT-II cells. The stimulation index refers to a fold-increase of the numbers of **(B)** CD44^+^, or **(C)** CD49^+^ CXCR3^+^ CD44^+^ OT-II cells compared to the media-only control. **(B,C)** Show 4–5 independent experiment repeats with SEM error bars, and statistical differences were calculated with a ratio paired *t*-test. *P* > 0.05; “ns,” not significant; ^*^P < 0.05; ^**^P < 0.01; ^***^P < 0.001; ^****^P < 0.0001.

Various approaches were used to elucidate the contribution to T cell priming and induction of lung homing molecules that was mediated by surface markers expressed at more than 3-fold higher levels on MedLN than ILN DCs. Firstly, specific Ab blockade was performed in a co-culture of *in vivo*-activated cytometry-sorted CD11b^+^ DCs and OT-II T cells. A significant reduction of the general activation of OT-II cells (Figure 5A) was detected after blockade of CD24, GL7, and using the anti-CD86 mAb control, but not anti-PDPN mAb. When the proportion of lung-homing CD49a^+^ CXCR3^+^ OT-II cells was analyzed (Figure S5A), there was a trend for CD24, GL7, and CD86 blockade to specifically suppress 30–40% of the induction of these cells.

Knowing that from the various surface markers tested on dLN CD11b^+^ DCs post-immunization, CD24 showed the highest average expression difference between the two LN sites (Figure 4D), we then delineated and characterized the CD24^+^ and CD24^−^ subsets in terms of their CD4^+^ T cell stimulation activity. A Principal Component Analysis that compared DCs from both LNs in terms of their combined expression of CD24, CD86, GL7, and PDPN (Figure S5B) also demonstrated that CD24 was a major contributing factor to the variation between MedLN and ILN DCs (PC1 loading matrix in Figure S4F). Therefore, CD24 appears to represent a good candidate to discriminate DCs elicited by i.n. vs. i.m. immunization, and a potential biomarker, in addition to CD86 and GL7, for DCs with high potency in terms of CD4^+^ T cell activation.

In the *in vitro* co-culture model of naïve OT-II with sorted Ag^+^ DCs from immunized mice, CD24^+^ CD11b^+^ DCs initiated higher levels of T cell stimulation (Figure 5B) compared to the CD24^−^ DC subset from the same dLN. There was no difference in the ability of CD24^+^ DCs from the draining MedLN and ILN for overall (CD44^+^) CD4^+^ T cell activation (Figures 5B,C). Using sorted DC subsets provided clearer results than the Ab blockade, by demonstrating that CD24^+^ CD11b^+^ DCs had an enhanced ability to selectively induce statistically greater numbers (Figure 5C), and a trend for higher percentages (Figure S5C), of *lung-homing* CD4^+^ T cells. Interestingly, CD24^+^ CD11b^+^ DCs from the MLN and ILN showed an equal capacity to prime T cells with a lung-homing phenotype (Figure 5C). While CD24^+^ and CD24^−^ CD11b^+^ DCs were sorted in these experiments, it is likely that the high co-expression of other surface molecules, such as CD86 and GL7 (Figures 4E,H) or possibly undefined secretory factors, also contribute to the enhanced induction of CD49a^+^ CXCR3^+^ OT-II cells by CD24^+^ DCs.

In addition to the use of lineage markers, such as CD4 and CD8, LN-resident DCs are often distinguished by low MHC-II and CCR7 expression, compared to migratory DC which express higher levels of MHC-II and CCR7, notably upon maturation (61–64). Therefore, to predict the anatomical origin of the potent CD24^+^ CD11b^+^ DCs, we evaluated their expression of CD4, MHC-II, and CCR7 after OVA immunization with either Poly(I:C) or CpG. Most CD24^+^ cDC were CD4^−^ (Figure S6A) and expressed higher CCR7 and MHC-II (Figures S6B,C), suggesting that this subset is not a cDC2 LN resident population and is likely to have migrated from the lung.

**Figure 6:**
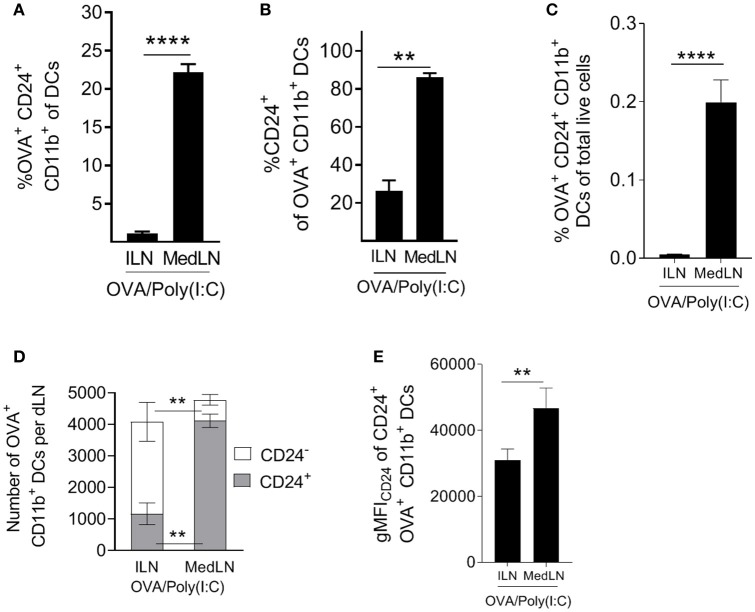
Intranasal immunization elicits higher frequencies and numbers of DCs with potent T cell priming capacity. **(A–E)** Conventional flow cytometric analysis of DCs from the dLN of mice immunized 1 day earlier by the i.n. or i.m. route with fluorescent OVA/Poly(I:C). **(A)** The percentage of OVA^+^ CD24^+^ CD11b^+^ DCs of total DCs, **(B)** the percentage of CD24^+^ cells of OVA^+^ CD11b^+^ DCs, **(C)** the percentage of OVA^+^ CD24^+^ CD11b^+^ DCs of total live cells. **(D)** Shows the number of CD24^+^ or CD24^−^ OVA^+^ CD11b^+^ DCs per dLN **(E)** shows the number of CD24^+^ or CD24^−^ OVA^+^ CD11b^+^ DCs per dLN. **(E)** Shows CD24 gMFI of OVA^+^ CD24^+^ CD11b^+^ DCs. **(A–E)** Show 4–7 independent experiment repeats with SEM error bars, and a paired *t*-test statistical comparison. ^**^P < 0.01; ^****^P < 0.0001.

Having established that CD24^+^ DCs are highly potent activators of CD4^+^ T cells, including lung-homing T cells, we evaluated the abundance of this subset *ex vivo* in the dLNs of immunized mice. Highly potent (OVA^+^ CD24^+^ CD11b^+^) DCs represented much higher proportions of total MedLN DCs compared to ILN DCs (Figure 6A; *P* = 0.0003). Furthermore, the majority (over 80%) of Ag^+^ CD11b^+^ DCs were CD24^+^ in the MedLN, whereas <30% were CD24^+^ in the ILN (Figure 6B), which directly corresponds to the overall CD24 MFI differences observed in the DC surface molecule screen (Figure 4D). The increased percentages of CD24^+^ DCs in the draining MedLN was not a TLR-targeting specific phenomenon because CpG and LPS also induced a similar distribution of Ag^+^ cells in the dLNs (Figure S6D). More importantly, analysis of the percentage of potent Ag^+^ CD24^+^ DCs of total live LN cells revealed a much higher density of this cell subset in the MedLN (Figure 6C), higher total numbers of Ag^+^ CD24^+^ CD11b^+^ per MedLN (Figure 6D), and higher expression of CD24 per cell (Figure 6E). Therefore, because the percentage of CD4^+^ T cells in both the MedLN and ILN is equivalent (Figure S6E), the ratio of potent CD24^+^ DCs per CD4^+^ T cell is much higher in the MedLN—which is likely to be a key contributing factor to the enhanced output of lung-homing T cells *in vivo* after i.n. compared to i.m. immunization.

## Discussion

Factors that shape the trafficking potential of T cells include tissue-specific cues, T cell receptor stimulation strength, as well as inflammation in secondary lymphoid organs and peripheral tissues (65). More specifically, soluble factors including vitamins and IL-12 are known to be involved in DC-mediated imprinting of gut and skin-homing molecules on T cells (21–23, 66). Apart from one report detailing how DC surface-bound TGF-β promotes the accumulation of CD8^+^ T cells in the lung (43), there is limited evidence of DC *surface* molecule-mediated induction of tissue homing molecules on T cells. Previous reports have shown that TCR signal strength, which relies on immunological synapse interactions at the cell surface, can skew the outcomes of T cell priming (67), for example, the balance between of effector vs. memory lymphocyte differentiation (57, 68). In the current study, surface molecules of *in vivo*-matured DCs were shown to be important mediators that control the quantitative output of the T cell response, as well as imprint lung-homing molecules on CD4^+^ T cells.

Priming the immune response via different routes *in vivo* resulted in different proportions of Ag-loaded and mature DC subsets in the corresponding dLNs. Importantly, we detected several site-specific differences in dLN DCs. Lung-associated LN DCs of immunized mice expressed higher levels of known costimulatory molecules than otherwise similar DC subsets in muscle-draining LNs. MedLN DCs were superior at overall priming of CD4^+^ T cells in terms of kinetics and output of CD44^+^ effectors, and notably induced rapid expression of lung-homing markers CXCR3 and CD49a. Rapid induction of tissue homing markers has been reported previously (69). This could therefore represent a means of deploying T cells to the mucosa within as little as 2 days post-priming, which is supported by *ex vivo* results detecting CD49a CXCR3 double-positive Ag-specific T cells in the dLNs of i.n. immunized mice at very early time-points post-immunization (Figure S2E).

A combination of *in vitro* and *ex vivo* data was used to infer the *in vivo* contribution of DCs to the induction of lung-homing of T cells. *Ex vivo* screening demonstrated that one of the most prominent differences between MedLN and ILN-derived CD11b^+^ DCs early after immunization was the level of CD24 expression. CD24 is a polyfunctional and widely-expressed molecule which is implicated in cell adhesion (70, 71), apoptosis and proliferation (72, 73). It also distinguishes Langerhans cells from dermal DCs (74), and has been used extensively as a lineage marker for B cell subsets (75, 76). The costimulatory property of CD24, when expressed by antigen presenting cells, is well-described (57, 73, 77), and one report details its specific contribution to inducing *mucosal* T cells and importance in DC subset classification (78). Subsequently, using sorted CD24^+^ and CD24^−^ CD11b^+^ DC subsets, we demonstrated that CD24^+^ DCs had an enhanced ability to prime CD4^+^ T cells *in vitro*, including those with a lung-homing phenotype. We then returned to quantify this potent DC subset *ex vivo*, showing that CD24^+^ CD11b^+^ DCs are found at much higher proportions in the draining LNs of i.n. compared to i.m.-immunized mice.

Recently, tissue-specific differences in surface molecule expression within major DC subsets have been observed at steady state in human tissues (79). Higher proportions of mature cDCs were detected in the draining LNs of human lung, and expressed relatively high levels of CD26 (79), a known T cell costimulatory molecule (80), compared to cDCs that drained other organs. The present study supports these findings by demonstrating the functional relevance of differential molecule expression patterns between anatomical sites. Namely, we show that DC surface marker signatures generated by different routes of immune priming result in quantitatively and qualitatively different CD4^+^ T cell responses.

Similarly to CD24, Ag^+^ CD11b^+^ MedLN DCs express >3-fold more PDPN and GL7, and to a lesser extent (1.5-fold) CD80 and CD86 than those from the ILN. Our results implicate CD24, CD86, and GL7 as DC surface molecules that contribute to T cell priming. GL7 has been used as an activation marker of lymphocytes (56), although to our knowledge its ability to function as a costimulatory molecule when expressed by DCs has not been described. Despite higher expression of PDPN in MedLN-derived DCs, this molecule did not appear to contribute to T cell priming, but was co-expressed with other costimulatory markers and therefore represents a surrogate marker of potent CD4^+^ T cell-activating DCs. In contrast, muscle LN-associated DCs expressed higher CD273 (PD-L2) and F4/80 and resulted in lower T cell responses compared to airway LN DCs, which corresponds to the known role of these markers in suppressing anti-microbial and anti-tumor T cell responses, or increasing regulatory T cell activity (81, 82).

Our results also demonstrate that CD24^+^ CD11b^+^ DCs are likely to be migratory (Figures S6A–C), which supports studies demonstrating that non-lymphoid tissue-resident DCs are potent inductors of T cell tissue-homing molecules for various tissues (23, 83–85). Furthermore, CD11b^+^ CD24^+^ CD64^low^ murine DCs have previously been shown to be functional homologs of human CD1c^+^ DCs (78), however the expression of CD24 on human DCs is not well-characterized at present. Nevertheless, the cDC2 subset constitutes the majority of conventional DC in all human tissues (79). cDC2 therefore represent an important cell subset to characterize fully, and a putative cellular target for vaccines and immunotherapies.

The immunization model used in the present study included the administration of soluble protein and TLR agonists, such as Poly(I:C), LPS, and CpG, via either the intranasal or intramuscular route. These routes differ greatly in terms of cellular and lymphatic structural features, as well as the microbiota and soluble microenvironment properties of the two sites, which could all contribute to the initiation of disparate immune responses after i.n. vs. i.m. immunization. Qualitative or quantitative differences in immune responses could also arise due to site-specific differences in TLR-mediated inflammation, or during the capture, drainage, and processing of Ag. Whether and to what extent these general features of the two immunization sites contribute to DC phenotype and subset density, or affect the imprinting of mucosal trafficking molecules on T cells independently to DCs, remains to be determined. We speculate that the different overall T cell priming capacity of ILN and MedLN-derived DCs is additive, i.e., relies on a combination of multiple co-stimulatory signals, both surface and secretory, as reviewed elsewhere (29, 86). Additional experiments are therefore needed to delineate the involvement of secretory factors and additional transcriptomic networks that are differentially regulated by DCs from different sites. As T cell-imprinting is also known to be affected by soluble factors produced in mucosal tissues (53), which can drain directly to dLNs via conduits (87), future research could test the effects of respiratory tract-enriched soluble factors, or CD24^+^ DC subset specific factors, for their contribution to the induction of T cell tissue homing receptors. Nevertheless, because the current study attempted to unravel *in vivo* mechanisms that modulate lung-homing T cell responses, we considered the inherent biases related to Ag delivery at different sites as necessary for the discovery of DC-related phenomena and processes that play a role *in vivo*.

Previous studies have determined that CCR4 (34) is upregulated on T cells primed by airway LN-derived DCs, which translates to increased lung-homing potential of T cells. T cell surface CCR4 upregulation was not detected in our model after DC co-culture (Figure S2B). This possibly stems from large differences in the study protocols regarding how the DCs were activated. The majority of experiments performed by Mikhak et al. included DCs that were Ag-loaded and activated *in vitro*, in contrast to the *in vivo* immunization used in the present study with a highly Th1-polarizing adjuvant (88). Except for a few isolated reports (89) and the present study, most previous studies have employed DCs that were loaded with Ag *ex vivo, in vitro*-generated, or Flt3L-expanded, often in the absence of stimuli to fully mature DCs, which could introduce artifacts, such as a skew toward regulatory, Th2, or anergic T cell responses. We therefore speculate that multiple different chemokine receptors, including CCR4, and those described in the present study, can all contribute to T cell homing to the lung in different contexts. This is supported by findings in the allergy and asthma research fields that demonstrate the involvement of CCR4 and other chemokine receptors in recruiting Th2-like cells to the lung (90, 91). In contrast, immunizations (18, 92, 93) or infections (94, 95) that induce Th1 or Th17-polarizing inflammation are known to prime lung-homing CD4^+^ T cells that express CCR6 and CXCR3.

Functional differences in cDC1 and cDC2 stem from their unique differentiation programs, and have been shown to greatly influence Ag-processing and presentation outcomes (96). CD103^+^ DCs are known to express very high levels of CD24 (68). These pulmonary CD103^+^ (CD24^high^) DCs are potent inducers of mucosal-homing CD8^+^ T cells (68), as well as mediators of IgA class-switch recombination (97). Placed in context with the current study, CD11b^+^ cDC2 expressed several fold less CD24 than their CD103^+^ cDC1 counterparts from the same tissue (data not shown), however, these CD24^+^ CD11b^+^ DCs provided completely sufficient co-stimulatory signals to induce a robust CD4^+^ T cell response including augmented numbers of cells with lung-homing capacity. The CD24 pathway could therefore be potentially targeted by immunostimulatory compounds with the aim of improving both CD4^+^ and CD8^+^ mucosal-homing T cell responses.

Lymphocyte trafficking to the desired tissue is thought of as a critical process in successful vaccination and immunotherapeutic strategies. One example of this comes from the pneumococcal field, where different immune stimuli, including infection and mucosal vaccination against respiratory pathogens in humans, have been shown to modulate tissue-homing receptor (α4β7 and L-selectin) expression of B cells (98). Here, our results link the density (Figures 1D, 6C) and phenotype (Figure 4E) of DCs in the respiratory tract LN with CD4^+^ T cell responses of greater magnitude and with greater lung-homing capacity (Figures 5B,C). As we focused on lung parenchyma homing in our model, future studies could be used to investigate whether the induction of CXCR3 and CD49a also favor the trafficking of T cells to other sites, including other mucosal tissues of the conducting airways, which are often critical entry points for pathogens.

Mucosal vaccination strategies are being investigated intensively (99–101), and represent a method to prime enhanced immune responses that protect the respiratory (102–106), reproductive (107), and gastro-intestinal tracts (108). However, mucosal approaches have undergone less clinical development to date compared to the parenteral route, possibly due to safety concerns about the intranasal route (109). Nevertheless, understanding the mechanisms that program T cell tissue-trafficking remains pertinent in the fields of vaccinology and immunotherapy. The current study and previous reports (34, 68, 97) put forward a strong rationale to administer vaccines in such a way that airway-resident DCs are loaded with Ag and matured. This could be achieved using either i.n. immunization, or alternatively direct intrapulmonary inhalation into the lungs via the mouth (110)—to avoid nasal administration of immune-stimulants within close proximity to the facial nerve (111).

Furthermore, inducing robust mucosal immunity to improve protection against respiratory pathogens might also be achieved using standard needle-based vaccination. For example, the development of next-generation parenteral adjuvants could aim to enhance the proportion of potent CD24^+^ DCs in muscle-draining LNs, or otherwise provide the costimulatory and secretory molecule signatures that enhance mucosa-homing CD4^+^ T cell responses. In fact, transcription factors, such as IRF4 that are critical for the development of pulmonary CD24^+^ CD11b^+^ DCs have already been characterized (112). In addition to enhancing lung-homing imprinting signals provided by DCs, there are also existing methodologies to increase total numbers of DCs that drain to the LN after parenteral immunization (113), which could address the lower ratio of DCs to T cells we observed in the muscle-draining LN.

To conclude, potent mucosal CD8^+^ T cell and humoral responses have been achieved using systemic immunization (114–116), which indicates the feasibility of improving parenteral injection-based vaccine strategies. However, the mechanisms involved have not been elucidated. Therefore, more in-depth knowledge of the molecular switches and signatures that influence T cell pulmonary-tract homing potential, which we have begun to unravel in the present study, paves the road for the rational design of novel and more effective immune prophylaxis.

## Data Availability

The datasets for this manuscript are not publicly available because this is the first study using a newly generated dataset, and the data can be made publicly available after publication. Requests to access the datasets should be directed to david.pejoski@unige.ch.

## Ethics Statement

Mice were bred or housed in pathogen-free animal facilities at the University of Geneva in accordance with local guidelines. Experiments were comprised of either all male or all female cohorts at 6–8 weeks of age, and conducted in accordance with the Geneva Veterinary Office Ethics Committee, and European guidelines.

## Author Contributions

C-AS, DP, P-HL, PA, and DC designed the study. The experiments were designed by DP and C-AS with input from MV, FA, and MB. The experiments were performed and analyzed by DP. The manuscript was written by DP and edited by C-AS, P-HL, PA, DC, MV, FA, and MB.

### Conflict of Interest Statement

The authors declare that the research was conducted in the absence of any commercial or financial relationships that could be construed as a potential conflict of interest.

## Abbreviations

dLN: draining lymph node
gMFI: geometric mean fluorescence intensity
ILN: inguinal lymph node
i.m.: intramuscular
i.n.: intranasal
i.v.: intravenous
MedLN: mediastinal lymph node
OT-II: OVA_323−339_ peptide specific CD4^+^ T cell
OVA: chicken ovalbumin protein
PDPN: podoplanin
*t*-SNE: *t*-distributed stochastic neighbor embedding.
