# Corrigendum: Site-Specific DC Surface Signatures Influence CD4^+^ T Cell Co-stimulation and Lung-Homing

**DOI:** 10.3389/fimmu.2019.02640

**Published:** 2019-11-11

**Authors:** David Pejoski, Marie Ballester, Floriane Auderset, Maria Vono, Dennis Christensen, Peter Andersen, Paul-Henri Lambert, Claire-Anne Siegrist

**Affiliations:** ^1^Department of Pathology and Immunology, Faculty of Medicine, University of Geneva, Geneva, Switzerland; ^2^World Health Organization Collaborating Center for Vaccine Immunology, Faculty of Medicine, University of Geneva, Geneva, Switzerland; ^3^Center for Vaccine Research, Statens Serum Institut, Copenhagen, Denmark; ^4^Department of Immunology and Microbiology, University of Copenhagen, Copenhagen, Denmark

**Keywords:** CD11b^+^ dendritic cells, lung CD4^+^ T cells, lung homing, tissue imprinting, costimulation, vaccination route

In the original article, there was a mistake in Figure 1A as published. The dot plot axis labels for the second leftmost dot plot were incorrect. The corrected Figure 1A appears below.

**Figure 1A:**
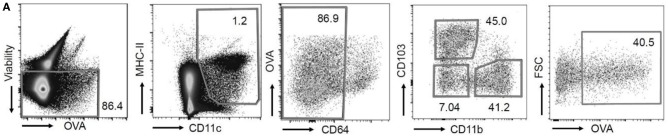


The authors apologize for this error and state that this does not change the scientific conclusions of the article in any way. The original article has been updated.

