# Supplementary material for: Site-Specific DC Surface Signatures Influence CD4+ T Cell Co-stimulation and Lung-Homing
Source: Front Immunol. 2019 Jul 18;10:1650. doi: 10.3389/fimmu.2019.01650 (PMC6668556; doi:10.3389/fimmu.2019.01650)
Supplement: Supplementary file 1 [file Data_Sheet_1.docx]

Supplementary Material


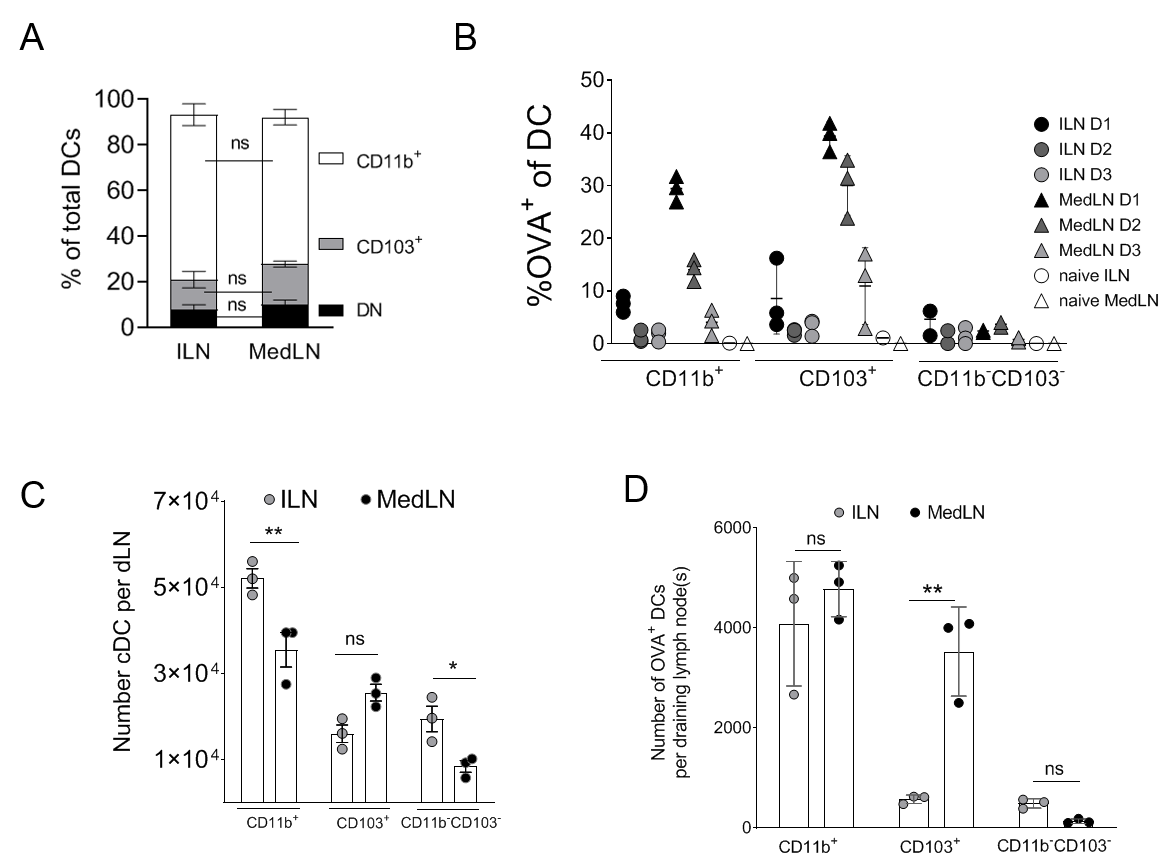


**Supplementary Figure 1.** **Percentages, total numbers, and timecourse of DC subsets in post-immunization dLNs.**  MedLNs or ILNs were harvested and pooled from naïve mice, stained for leukocyte lineage markers, and evaluated by flow cytometry using the gating strategy shown and described in **Figure 1A**. (**A**) shows the percentage of the indicated DC subsets of total LN DCs from naïve mice. (**B**) shows the percentage of OVA^+^ cells within the dLN CD11b^+^ DC compartment at the indicated time-points (day 1; D1, day 2, D2, day 3, D3) after fluorescent OVA/Poly(I:C) immunization via the i.m. or i.n. routes, or in naïve mice. One of 3 representative experiments shown with 1-3 individual mice per immunization route and S.D. error bars. (**C**) the absolute numbers of DC subsets per dLN or **(D)** the number of OVA^+^ DCs per dLN, at 24h post-immunization with fluorescent OVA/Poly(I:C). LNs from 3-12 mice per route were pooled, representing 3 or more independent experiments and the SEM. Statistical differences were calculated using a one-way ANOVA with post-test as described in the Materials and Methods. P > 0.05; ‘ns’, not significant, P < 0.05; *, P < 0.01; **.


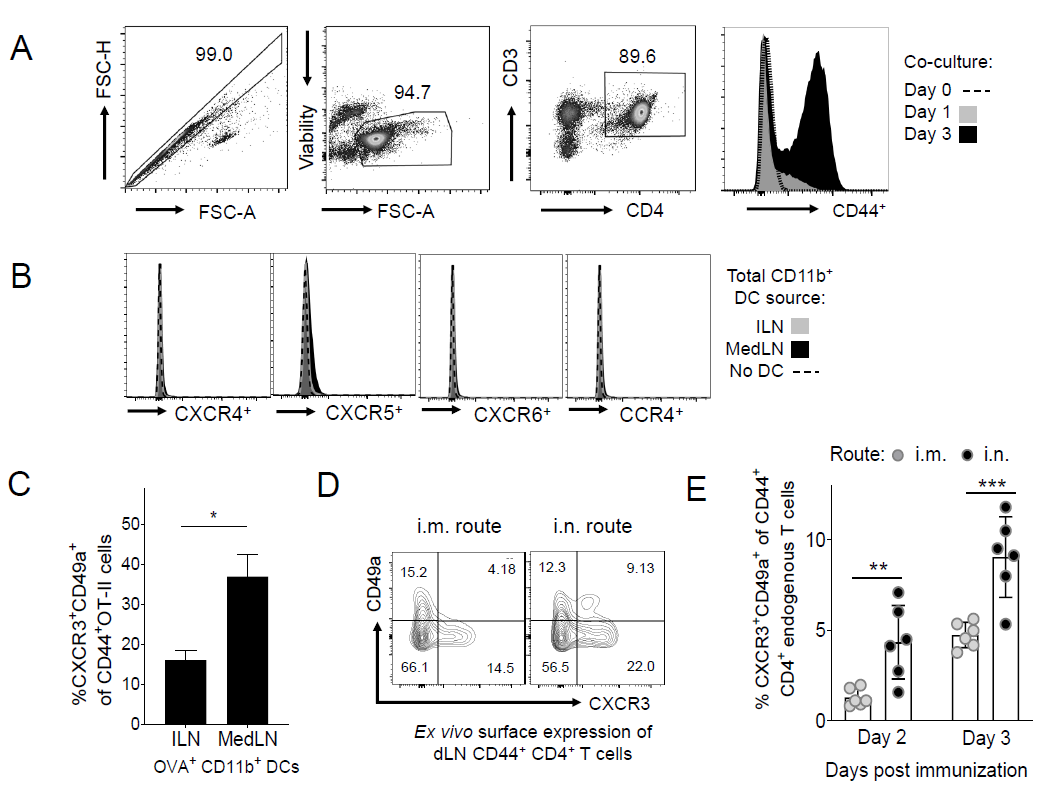


**Supplementary Figure 2.** **OT-II cell and endogenous *ex vivo* T cell analysis after co-culture with DCs.** (**A**) shows the gating strategy used to exclude doublets, dead cells, CD3^-^ CD4^-^ , and select CD44^+^ (activated) cells from OT-II CD4^+^ T cells, at day 0, 1, or 3 of co-culture with total CD11b^+^ DC subsets from the draining LN of immunized mice (MedLN representative data shown). After 3 days of co-culture with sorted DCs or media alone, OT-II cells were stained for the indicated markers and acquired on a flow cytometer to evaluate surface expression as (**B**) histograms of the dLN, representative of at least three experimental repeats after incubation with total CD11b^+^ DCs, and (**C**) the percentage of cells that expressed both lung homing markers (CD49a and CXCR3) within the CD44^+^ OT-II gate, after incubation with OVA^+^ CD11b^+^ DCs. **(D)** and **(E)** BL6 mice were immunized and the dLNs were analysed directly *ex vivo* by flow cytometry to determine the percentages and phenotype of endogenous lung homing T cells. Representative gating for lung homing markers (CD49a and CXCR3) within the CD44^+^ CD4^+^ T cell gate is shown in **(D)** at 3 days post-immunization, and in **(E)** the bars represent the mean pooled from 2 experiments at the indicated time-points post-immunization, with individual mice shown as separate points, with SD error bars. P < 0.05, *; P < 0.01; **, P < 0.001; ***.


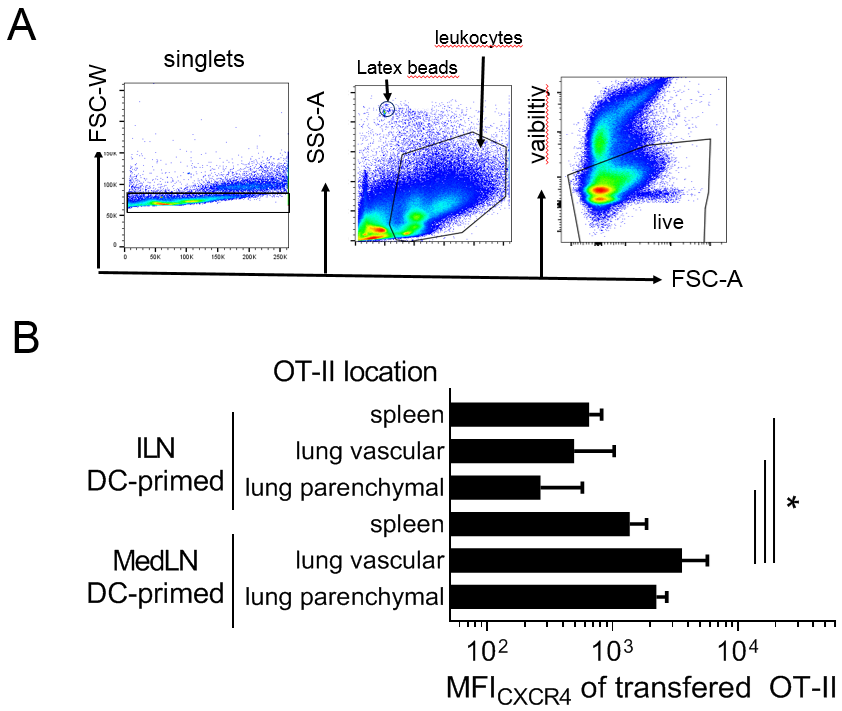


**Supplementary Figure 3.** **Preliminary gating strategy and additional *ex vivo* homing marker analyses of adoptively transferred OT-II cells** (**A-B**) OT-II cells were activated *in vitro* via co-culture for 3 days with sorted MedLN or ILN CD11b^+^ DCs from i.n. or i.m. immunized mice, then transferred into naïve Rag2-/- mice (n = 6 per DC co-culture type). After 18 hours, the indicated organs of these recipient mice were removed and characterized by flow cytometry, as described in (**Figure 3**) and the Materials and Methods. The preliminary steps of the flow cytometry gating strategy shown in (**A**) were used to exclude debris, remaining red blood cells, and dead cells, followed by the gating strategy shown in the Results Section (**Figure 3B**). Cell numbers in each sample could be accurately calculated using latex bead controls that were added during the staining procedure. (**B**) shows the expression levels of CXCR4 of activated OT-II cells in recipient Rag2-/- mice at 18 hours post transfer, which were compared using a one-way ANOVA with post-test of pooled data from two independent experiments. P < 0.05; *.


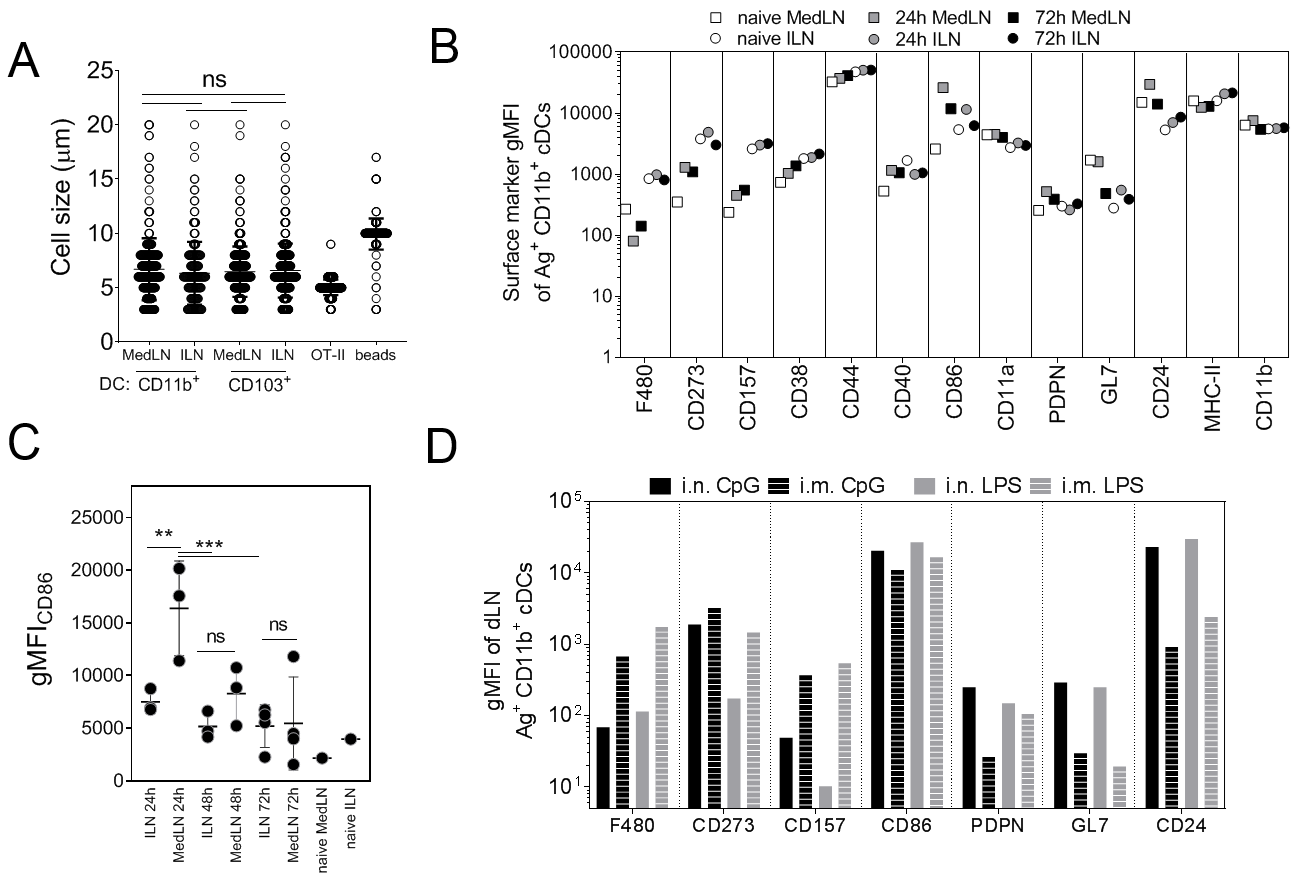

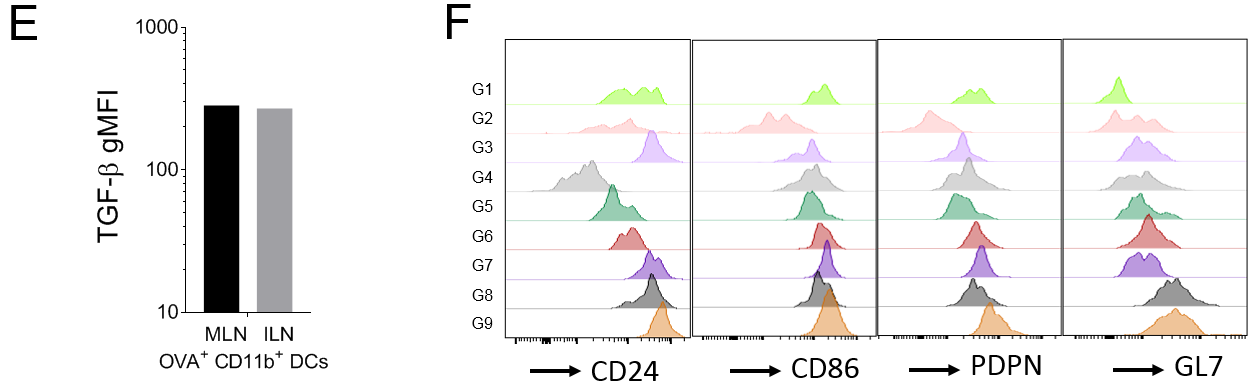


**Supplementary Figure 4.** **Absolute size and surface marker expression kinetics of DC subsets from different draining LNs.** Mice were immunized by the i.n. or i.m. route with fluorescent OVA/Poly(I:C) and dLNs were harvested 24h later and either stained, sorted, or rested as indicated. (**A**) DC subsets were sorted and rested overnight in complete media. The graph shows the absolute size of DCs as measured by an image based cytometer. Each image acquisition contained 200 to 375 cells or control beads per sample, and data represents one of two independent experiments. (**B-E**) Mice were immunized by the i.n. or i.m. route with fluorescent OVA/Poly(I:C) and dLNs were harvested at 24h (**C, D, E, F**), 48h (**C**), or 72h (**B** and **C**) post immunization as indicated, and these figures show the net gMFI of the indicated surface markers of OVA^+^ CD11b^+^ DCs for (**B**, **D**, **E**) pools of draining LN suspensions, or (**C**) individual mice. One of two representative experiments shown. In (**C**), S.D. error bars, and statistical differences were calculated using a one-way ANOVA with post-test. (**D**,**E**) represent one of two independent experiments with similar results. (**F**) shows histograms of individual marker expression for each of the 9 t-SNE gates described in (**Figure 4**). P > 0.05; ‘ns’, not significant, P < 0.05; *, P < 0.01; **, P < 0.001; ***.


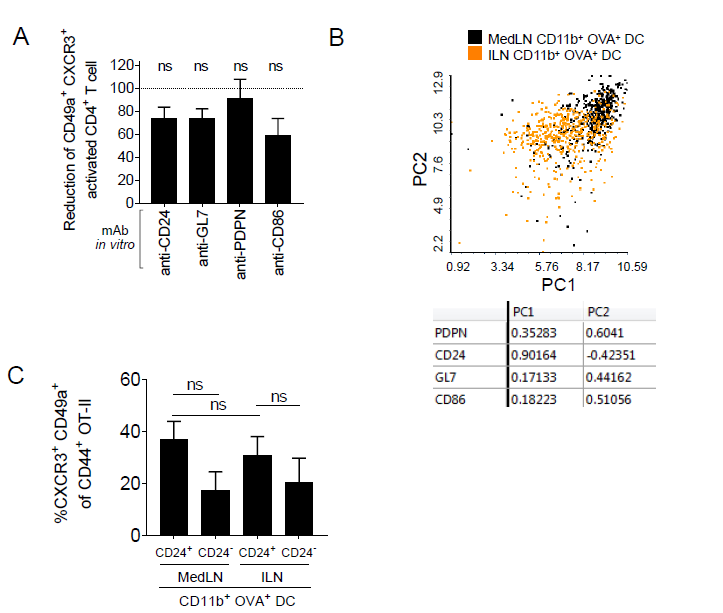


**Supplementary Figure 5.** **Contribution of individual DC surface markers to the activation of lung homing T cells *in vitro*, and also to LN site-specific molecular signatures** (**A**) shows the effect of incubating mAbs with OVA^+^ CD11b^+^ DCs sorted from the draining MedLN one day after i.n. immunization, on *in vitro* activation of OT-II that co-express lung homing markers CXCR3 and CD49a, compared to after DC incubation with isotype control Abs. A single sample t test was used to determine whether the reduction was significant compared to the expected value of 100% for the isotype control. (**B**) shows a Principal Component Analysis plot and its associated component loading matrix, based on equal numbers of OVA^+^ CD11b^+^ DCs from the ILN and MedLN. Data represents one of two independent experiments with similar results. (**C**) shows the percentages of CXCR3^+^ CD49a^+^ cells within the CD44^+^ gate of OT-II cells after 3 days of co-culture with either cytometry-sorted CD24^+^ or CD24^-^ OVA^+^ CD11b^+^ DCs from the dLNs of mice that had been immunized 24h earlier with fluorescent OVA/Poly(I:C) via the i.m. or i.n. route. (**A** – **C**) Data is representative of at least 3 independent experiments, P > 0.05; ‘ns’, not significant.


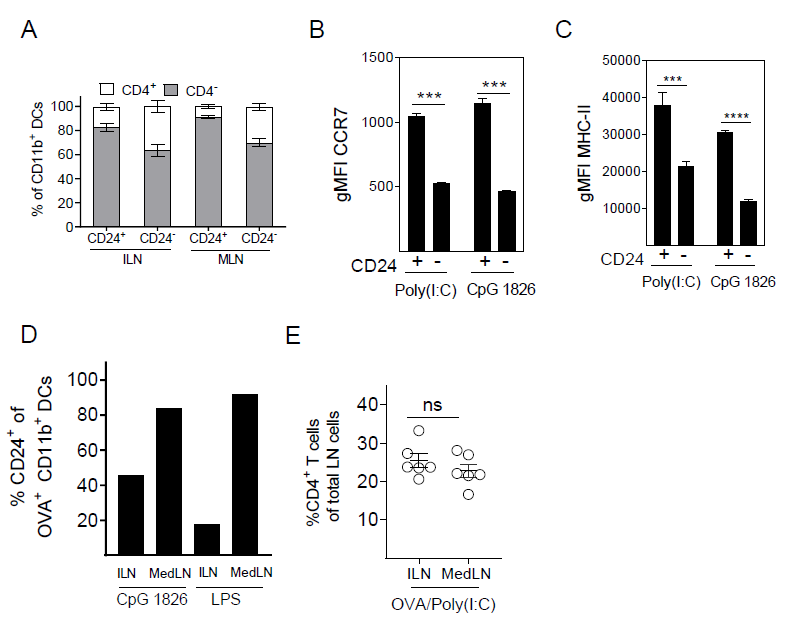


**Supplementary Figure 6.** **Flow cytometric characterization of dLNs DCs or CD4^+^ T cells *ex vivo* post-immunization.** (**A-E**) shows the percentage or surface marker MFI of DC subsets from the indicated dLN (ILN or MedLN) of mice immunized 24 hours prior via the i.m. or i.n. route with fluorescent OVA adjuvanted with either Poly(I:C), CpG 1826, or LPS as indicated. (**A**) the percentage of CD4^+^ or CD4^-^ DCs within CD24^+^ or CD24^-^ CD11b^+^ DC subsets, with 3 mice per group and SD error bars. CCR7 (**B**) and MHC-II (**C**) geometric MFIs of CD24^+^ or CD24^-^ CD11b^+^ DC populations were determined using pooled LNs of 3 – 6 mice that were immunized 24h earlier. (**D**) The percentage of CD24^+^ cells on pre-gated OVA^+^ CD11b^+^ DCs in the draining LN of mice immunized with fluorescent OVA and either CpG or LPS adjuvants, showing results from pooled draining LNs of at least 3 mice. (**A-D**) data is representative of 2 – 3 independent experiments. (**E**) the percentage of CD4^+^ T cells of total live dLN cells at 24h post-immunization, symbols represent the mean percentage from individual experiment repeats using pooled LNs from 3 – 12 mice per route per experiment, and compared using a ratio paired t test. P > 0.05; ‘ns’, not significant, P < 0.001; ***, P < 0.0001; ****.
